# Cell phone ownership and modern contraceptive use in Burkina Faso: implications for research and interventions using mobile technology

**DOI:** 10.1016/j.contraception.2018.11.006

**Published:** 2019-03

**Authors:** Abigail R Greenleaf, Saifuddin Ahmed, Caroline Moreau, Georges Guiella, Yoonjoung Choi

**Affiliations:** a Department of Population Family and Reproductive Health, Johns Hopkins University, 615 N. Wolfe St., Baltimore, MD 21205, United States; b Gender, Sexual and Reproductive Health, Centre for Research in Epidemiology and Population Health, (CESP), F-94805, Villejuif, France; c Institut Supérieur des Sciences de la Population, University of Ouagadougou, Institut Supérieur des Sciences de la Population, Ouagadougou, Burkina Faso

**Keywords:** Cell phones, mHealth, Africa south of the Sahara, Burkina Faso, Family planning, Performance Monitoring and Accountability 2020

## Abstract

**Objectives:**

With over 420 million unique cell phone subscribers in sub-Saharan Africa, the opportunities to use personal cell phones for public health research and interventions are increasing. We assess the association between cell phone ownership and modern contraceptive use among women in Burkina Faso to understand the opportunity to track family planning indicators using cell phone surveys or provide family planning interventions remotely.

**Study design:**

We analyzed data from a cross-sectional, nationally representative population-based survey of women of reproductive age in Burkina Faso, the Performance Monitoring and Accountability 2020 Round 4, which was conducted between November 2016 and January 2017.

**Results:**

Among the 3215 female respondents aged 15 to 49 years, 47% reported cell phone ownership. Overall, 22% of women reported current modern contraceptive use. Women who owned a cell phone were more likely to report modern contraceptive use than those who did not (29% versus 15%). Adjusted for covariates (age, wealth, education, area of residence and marital status), the odds of reporting modern contraceptive use were 68% higher among cell phone owners compared to nonowners (odds ratio=1.68, 95% confidence interval 1.3–2.1). Method mix was substantially more diverse among those who owned cell phones compared to their counterparts.

**Conclusions:**

The study shows that cell phone ownership is significantly associated with modern contraceptive use in Burkina Faso, even after adjusting for women's sociodemographic characteristics. These results suggest that cell phone ownership selectivity and associated biases need to be addressed when planning family planning programs or conducting surveys using cell phones.

**Implications:**

Cell phones can be used for myriad family planning purposes, from confidential data collection to contraceptive promotion and knowledge dissemination, but ownership bias is significant. A cell-phone-based intervention or population-based survey is unlikely to reach a critical mass of the population at highest risk for unintended pregnancy.

## 1 Background

The fastest growing cell phone market in the world is sub-Saharan Africa (SSA). In 2016, there was a 43% mobile penetration rate in the region, and by 2020, half of the population is projected to have cell phone service [1]. Urbanization, expansion of cell phone network coverage and the decreasing cost of purchasing a cell phone have contributed to increased cell phone ownership throughout SSA [2], [3]. Greater cell phone ownership presents the opportunity to communicate with respondents remotely for myriad public health purposes: to collect survey or surveillance data, improve medical adherence and send appointment reminders or conduct behavioral change interventions [4], [5], [6], [7]. Mobile health (mHealth) project growth has been concomitant to increased cell phone ownership. The evidence base for mHealth initiatives is still solidifying [8], [9], but practitioners hope that mHealth can help ameliorate infrastructural deficiencies, shortage of health care workers and problems in reaching remote populations [10].

SSA has also seen growing national and international efforts to expand access to family planning services in response to rapid population growth and high unmet need [11]. Sexual and reproductive health programs in SSA using mHealth have primarily focused on behavior change communication programs, sharing family planning knowledge through short message service [12], either among a general population or for a targeted audience of adolescents [13], [14], [15], [16]. The use of mHealth for population data collection is still relatively rare in SSA, and no studies to date have used remote data collection to estimate family planning indicators at the population level [17]. However, the applications are emerging: a recent study in Ivory Coast piloted a random digit dial survey about HIV risk behaviors [18].

This study focuses on Burkina Faso, where both cell phone penetration and modern contraceptive use rapidly increased in recent years. The ambitious national family planning goal is to increase the modern contraceptive rate among married women to 32% by 2020 [19]. mHealth programs in Burkina Faso are nascent; an mHealth program in the Northwest region used interactive voice response to remind community health workers to prompt pregnant women and people living with HIV to attend appointments [20]. The use of cell phones in family planning interventions and data collection at the population level, however, raises issues of selective ownership and its implications in reaching target populations [5], [21]. In Burkina Faso, while 86% of households [22] and 43% of individuals owned their own cell phones [1], phone ownership is biased towards men, and educated and urban populations [3].

The objective of this research is to (a) understand the association between cell phone ownership and modern contraceptive method use among women of reproductive age and (b) assess differential method mix among modern method users by cell phone ownership in Burkina Faso using a nationally representative survey. Study findings will inform future use of mHealth for family planning program delivery or monitoring in the country and the West African region by characterizing selection bias and its implications.

## 2 Methods

### 2.1 Data

We analyze data from the Performance Monitoring Accountability 2020 (PMA2020) fourth round survey collected in Burkina Faso. A network of locally trained female resident enumerators collects the nationally representative data. The resident enumerators administer face-to-face interviews, recording data on cell phones to track key family planning indicators under the Family Planning 2020 initiative, which aimed to enable 120 additional million women to use contraceptives by the year 2020 [23]. Since 2013, PMA2020 surveys have been conducted every 6 to 12 months in 11 countries (http://www.pma2020.org). The surveys in Burkina Faso use a two-stage stratified cluster design, starting with a selection of geographical sample clusters based on probability proportional to size in each of the urban and rural strata followed by a random selection of households within each cluster. Detailed sampling methods and procedures are available elsewhere [24].

The surveys include household and female interviews. Household interviews collect basic demographic data on all household members and household characteristics. Within each sampled household, all women 15 to 49 years old are eligible for the interview after their informed consent. The female survey collects information on women's background characteristics, contraceptive use and reproductive health. Regarding contraception, women are asked if they have ever heard of each available contraceptive method in the country. Then, women are asked if they (or their partner) are currently using anything to prevent or delay pregnancy. Women who report using a method are further asked to specify the type of methods currently used.

In Burkina Faso, PMA2020 has collected five rounds of nationally representative data since 2014. The fourth survey was conducted between November 2016 and January 2017. A total of 2751 households and 3215 eligible women were interviewed. The response rates were 95.4% for the household interview and 95.4% for the female interview [25]. Ethical approval was obtained from the Johns Hopkins Bloomberg School of Public Health's Institutional Review Board as well as the *Comité d'éthique pour la recherche en santé* in Ouagadougou, Burkina Faso. Datasets are available to the public for research purposes [24], [26].

### 2.2 Measures

The key dependent variable in this analysis is reported current use of modern contraceptive method(s). Modern methods, as defined by the World Health Organization, include pills, implants, injectables, intrauterine device, condoms, female and male sterilization, lactational amenorrhea method, emergency contraception and standard days method [27]. Women are categorized into those using any modern method vs. nonusers of modern methods.

The key independent variable is cell phone ownership. Women were asked “How many phone numbers do you have?” and were considered cell phone owners if they reported having one or more phone numbers.

Other independent variables include women's sociodemographic characteristics such as age, which was categorized into four groups (15–19, 20–29, 30–39, 40–49), current union status (in union, i.e., currently married or living with a partner, vs. not in union), residential area (urban vs. rural), highest school ever attended (none, primary, or secondary and higher), household wealth (lowest quintile, three middle quintiles or highest quintile), and having electricity (yes vs. no). These variables were selected based on literature reviews and conceptual frameworks on the determinants of contraceptive use. Although electricity is typically used to calculate household wealth index, in this analysis, it was considered as an additional covariate because cell phone use requires access to electricity.

### 2.3 Analysis

The current analysis includes 3215 women aged 15 to 49 years who completed the PMA2020 Round 4 survey. After conducting descriptive analyses for the distribution of the key variables, we examined bivariate associations between women's sociodemographic characteristics and cell phone ownership as well as modern contraceptive use with *χ*^2^ tests adjusted for the complex survey design. To estimate odds of modern contraceptive use by cellphone ownership, we conducted logistic regression analysis using bivariate and multivariable models. All covariates significantly related with the outcome in the bivariate analyses were included in the multivariable model. Analyses were adjusted for sampling weights and survey design, which account for two-stage cluster sampling and nonresponse rates [28].

## 3 Results

### 3.1 Characteristics of sample and cell phone ownership

Study sample characteristics are presented in Table 1. Overall, women were on average 28.9 years old, 70% were married, and 79% had children. Three quarters of the sample lived in rural areas, and only a third of women had ever attended school. Approximately 47% of respondents reported cell phone ownership, while 89% of households had cell phones. Women's cell phone ownership was positively associated with wealth, education and living in an urban area (Table 1). Among cell phone users, 83% reported only one number, 14% had two numbers, and the remaining 3% had three or more phone numbers*.* The mean cell phone numbers among cell phone owners was 1.2.

**Table 1:** Characteristic of study sample and cellphone ownership by background characteristics (*n*=3215).

	% Distribution among total population	% Distribution of cell phone ownership by background characteristic		Rao and Scott p value^a^
		Owner	Not an Owner	
Total		46.9	53.1	
Age (years)				
15–19	22.1	39.9	60.1	
20–29	34.6	51.1	48.9	
30–39	26.9	48.5	51.5	
40–49	16.4	44.9	55.1	<.001
Parity				
Ever given birth	78.8	52.3	47.7	
Never given birth	21.2	47.4	52.5	.16
Urban/rural				
Urban	24.4	71.9	28.1	
Rural	75.6	38.9	61.1	<.001
Marital status				
Currently not in union	30.5	50.6	49.4	
Currently in union	69.5	45.3	54.7	.17
Highest school attended				
Never	64.5	40.4	59.6	
Primary	16.1	55.2	44.8	
Secondary or higher	19.4	71.1	28.9	<.001
Household wealth (quintile)				
Lowest	21.5	30.3	69.7	
Lower	19.1	37.0	63.0	
Middle	20.7	40.0	60.0	
Higher	17.4	51.1	48.9	
Highest	21.3	75.9	24.1	<.001
Household electricity				
Yes	60.0	59.4	40.5	
No	40.0	38.5	61.5	<.001

Note: % estimates are adjusted for sampling weight.

a p value for Rao and Scott's *χ*^2^ test for differential distribution of cell phone ownership by background characteristics.

### 3.2 Cell phone ownership and modern contraceptive use

Among all women interviewed, 22% reported current use of a modern method. Modern contraceptive use was significantly higher among women who owned cell phones: 29% of cell phone owners used modern contraception versus 15% of nonowners.

All covariates were significantly associated with modern contraceptive use in bivariate analyses except being in the lowest quintile (Table 2). Specifically, cell phone owners had twice the odds of using modern contraception compared to nonowners in the bivariate analysis [odds ratio (OR) 2.26, 95% confidence interval (CI) 1.74–2.94]. The positive association remained significant in multivariable analysis, with 68% higher odds of modern contraceptive use among cell phone owners compared to nonowners (OR 1.68, 95% CI 1.29–2.20), adjusting for sociodemographic characteristics. We further assessed interactions between key background characteristics and cell phone ownership for assessing whether the relationship of cell phone ownership and modern contraceptive use varies by background characteristics and found significant interaction by women's educational level. Specifically, the association between cell phone ownership and modern contraceptive use was greater among women with higher education. In a stratified analysis, among women with secondary or higher education, cell phone ownership was associated with four times higher odds of using a modern method (OR 4.0, 95% CI 1.9–8.4), whereas there was no statistically significant difference in modern method use between cell phone owners and nonowners among women with no education (OR 1.3, 95% CI 0.9–1.8).

**Table 2:** Modern contraceptive use by cell phone ownership and background characteristics: OR based on bivariate and multivariable logistic regression analyses (*n*=3215).

	Bivariate	Multivariable^a^
	OR (95% CI)	OR (95% CI)
Cell phone ownership		
No (reference)		
Yes	2.26 (1.74–2.94)	1.68 (1.29–2.20)
Age (years)		
15–19 (reference)		
20–29	3.36 (2.41–4.67)	2.93 (1.97–4.37)
30–39	3.57 (2.46–5.20)	3.22 (2.02–5.15)
40–49	2.01 (1.33–3.06)	1.98 (1.20–3.29)
Marital status		
Currently not in union (reference)		
Currently in union	1.70 (1.30–2.23)	1.74 (1.23–2.45)
Residential area		
Rural (reference)		
Urban	2.10 (1.64–2.76)	1.28 (0.94–1.75)
Highest school attended		
No education (reference)		
Primary	1.72 (1.30–2.26)	1.69 (1.28–2.22)
Secondary or more	1.46 (1.08–1.98)	1.56 (1.13–2.18)
Household wealth		
Lowest quintile	1.08 (0.71–1.62)	1.21 (0.79–1.86)
Middle three quintiles (reference)		
Highest quintile	2.48 (1.91–3.23)	1.66 (1.20–2.31)

Note: Analyses adjusted for sampling weight.

a The multivariable model included all covariates presented in the table.

### 3.3 Method mix among modern method users by cell phone ownership

**Fig. 1:**
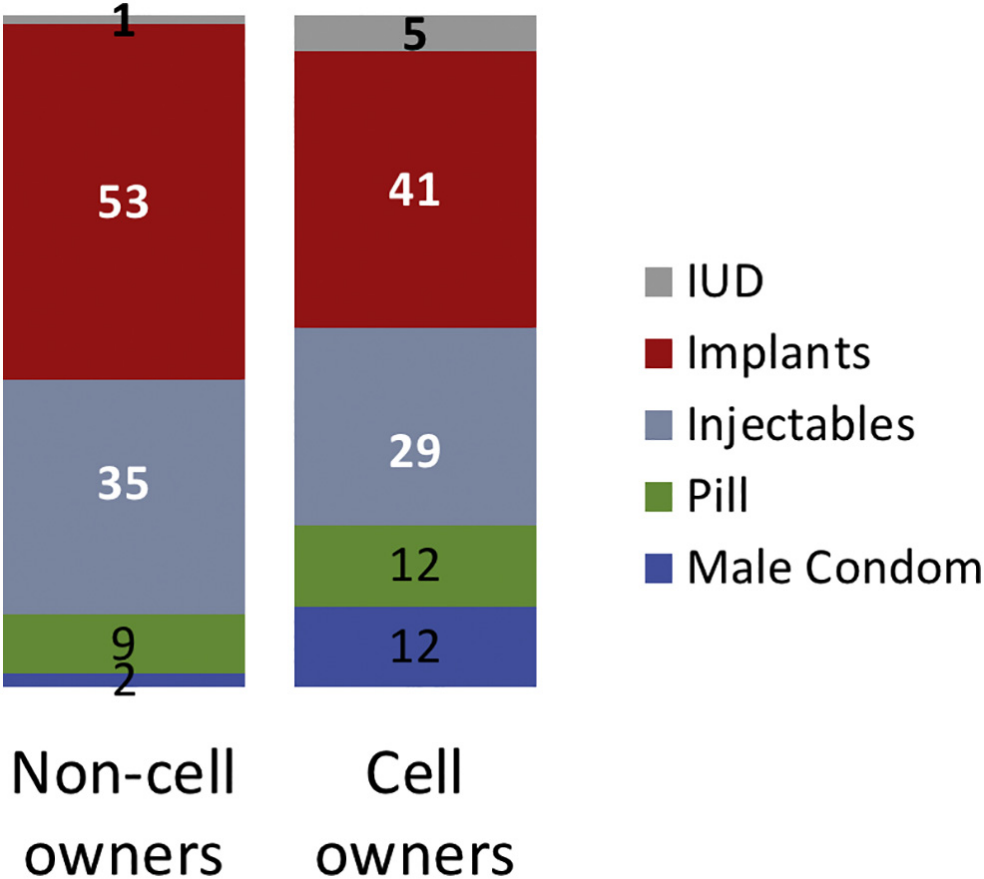
Modern method mix among current users by cell phone ownership status (%). Note: % estimates are adjusted for sampling weight.

Two methods — implants and injectables — accounted for 88% of modern method use among nonowners (Fig. 1). Cell phone owners, however, had a more diverse method mix, with implants and injectables accounting for 71% of modern method use, while male condoms, pills and IUDs accounted for 28% of the modern method mix. Overall, the order of prevalent methods was the same among cell phone owners and nonowners. When looking at cell phone ownership by type of modern method used (Table 3), implants users were the most evenly split between nonowners (41%) and cell phone owners (59%). The greatest differential was seen among male condom users: 7% of male condom users did not own cell phone, whereas 93% did. Finally, 60% of injectable users and 70% of pill users owned a cell phone.

**Table 3:** Cell phone ownership by type of modern method^a^ (%).

	Non cell phone owner	Cell phone owner
IUD (*n*=40)	11	89
Implants (*n*=322)	41	59
Injectables (*n*=207)	40	60
Pill (*n*=115)	30	70
Male condoms (*n*=104)	7	93
Total (*n*=788)	36	64

Note: % estimates are adjusted for sampling weight. The number of women using each method is unweighted.

a The most effective method currently used, if multiple methods were reported.

## 4 Discussion

This study examined the association between cell phone ownership and modern contraceptive use in Burkina Faso. Approximately half of female population owned cell phones, and the ownership was higher among socioeconomically advantaged groups. Women with cell phones had 68% higher odds of using a modern contraceptive compared to women with no cell phone, adjusting for sociodemographic characteristics. Women who did not own phones, who were poorer or who had no or little education were least likely to use modern contraception. A substantial proportion of short-acting hormonal method users, who may be eligible for mHealth interventions for a refill reminder, did not own a cell phone (40% and 30% among injectable and pill users, respectively).

Employing nationally representative data, this study examined cell phone ownership and contraceptive use in a setting where cell phone ownership is rapidly increasing. A study in Nigeria assessed the relationship between maternal health service utilization, including contraceptive use, and cell phone ownership — but focused on women who had children under 2 years of age, who tended to be younger than overall women of reproductive age, in five states in the country. Nonetheless, their results were consistent with our findings — women who did not own a phone had half the odds of using modern contraception compared to women who owned a phone [29].

Of note, we measured women's ownership of cell phones instead of household ownership. Cell phone ownership has typically been collected at the household level by national surveys. Studies examining cell phone ownership or access among females in SSA are relatively rare. The scant literature reveals a substantial ownership difference by gender: cell phone ownership data from Niger indicates a gender gap by 45% points [30]. In our study population, almost 90% of households had cell phones, but less than half of women reported owning a cell phone.

Limitations of this study include measurement error about women's cell phone ownership. Women may have reported a family member's phone number rather than their personal phone number. This potential misreport has implications for cell phone survey participation, as husbands may act as gatekeepers by controlling their spouses' access to phones. More importantly, cell phone ownership does not necessarily translate into an operational phone due to poor network service and/or intermittent electricity. As cell phone network coverage expands, however, the number of women who will become reachable by phone will grow. Also, with the rapid increase in phone ownership, our findings may have limited relevance in the near future in Burkina Faso. Although the data were collected in November 2016, changes in the profile of phone owners are likely to occur even in a short time frame. The value of owning a cell phone is well understood by women [30]; thus, we expect increases in levels of ownership among women, albeit growth will be slowest among poorest women. Finally, while differential contraceptive practices by cell phone ownership identified potential limitations of mHealth programs in reaching women in general, we did not assess its implications in reaching women with unmet need for family planning. With still relatively low modern contraceptive prevalence in Burkina Faso, demand for family planning may correlate strongly with socioeconomic characteristics and, thus, cell phone ownership.

## 5 Conclusion

In Burkina Faso, women with cell phones are more urban, educated and economically advantaged, and they have about 70% higher odds of using modern contraception compared to women without cell phones. In addition, among modern method users, those who do not own a cell phone tend to primarily use implants and injectables. Among short-acting hormonal method users who may be eligible for mHealth refill reminders, a substantial portion do not own a cell phone. The findings provide programmatic implications for family planning mHealth interventions as well as research based on cell phone survey data.
